# Using size-weight relationships to estimate biomass of heavily targeted aquarium corals by Australia’s coral harvest fisheries

**DOI:** 10.1038/s41598-023-28447-w

**Published:** 2023-01-26

**Authors:** Kai I. Pacey, Ciemon F. Caballes, Morgan S. Pratchett

**Affiliations:** 1 Australian Research Council Centre of Excellence for Coral Reef Studies, James Cook University, Townsville, QLD 4811 Australia; 2 National Science Foundation Established Program to Stimulate Competitive Research-Guam Ecosystems Collaboratorium for Corals and Oceans, University of Guam-Marine Laboratory, Mangilao, Guam, 96923 USA

**Keywords:** Environmental impact, Marine biology, Animal physiology, Conservation biology

## Abstract

Coral reefs are highly threatened environs subject to ongoing unprecedented degradation as a result of anthropogenic activities. Given the existential threat to coral reef ecosystems, extractive industries that make use of coral reef resources, are facing significant public and political pressure to quantify and justify their environmental impact. In Australia, hundreds of thousands of live scleractinian (hard) corals are harvested annually directly from the wild to supply the growing international marine aquarium trade. Many of the most popular and high value aquarium corals are believed to be slow growing, which would make them particularly vulnerable to over-fishing. Corals present a number of unique challenges for fisheries management, not least of which, is the marked variation in the size of corals, which may be harvested in whole or in part. This issue is further compounded because harvest limits are typically weight-based, but there is very limited information on the standing biomass of corals in targeted stocks. Herein, we describe size-weight relationships for some of Australia’s most heavily targeted coral species (*Catalaphyllia jardinei*, *Duncanopsammia axifuga*, *Euphyllia glabrescens*, *Homophyllia* cf. *australis*, *Micromussa lordhowensis*, *Trachyphyllia geoffroyi*), which allows estimation of standing biomass from transect surveys. This work represents an important first step in the development of ecologically sound management strategies by bridging the gap between catch reporting and stock assessments.

## Introduction

Marine aquarium fisheries supply an international trade involving the collection and sale of millions of live organisms^1^, many of which are collected from the wild in countries such as Indonesia, Fiji, and Australia^1,2^. Coral harvest fisheries supply a major component of the aquarium trade in the form of live aquarium coral specimens, the vast majority of which are destined for home or public aquaria^2,3^. Coral harvesting for the marine aquarium trade is highly selective, and involves hand collection of select coral colonies or fragments^4,5^. Therefore, the direct and independent ecological impacts of coral harvest fisheries are generally regarded as very minor (e.g., see Harriott^4^), especially compared to the myriad of large-scale disturbances (e.g., outbreaks of coral predators and climate-induced bleaching) that are affecting wild coral stocks. However, the widespread and accelerating degradation of coral reef ecosystems^6,7^ and coastal modification^8,9^ may undermine the sustainability of coral harvest fisheries. In light of the already concerning state of coral reef environments globally, industries that access or use coral reef environments, such coral harvest fisheries, and the governmental and intergovernmental bodies that manage them, are currently experiencing significant and increasing public, economic, and political pressure to deliver scientifically defensible and ecologically relevant management policies^10–12^.

All hard corals are listed in Online Appendix II of the Convention on the International Trade in Endangered Species (CITES), and strict stipulations must be met for the trade of listed wildlife to be considered legal in all 184 signatory countries. To supply the growing international demand for live aquarium coral species, hundreds of thousands of coral pieces are traded annually valued at millions of dollars. Wood et al.^1^ examined global import reports from the CITES trade database, noting marked increases in the volume of trade from ~ 600,000 pieces in 2000 to ~ 990,000 pieces in 2010, peaking at > 1,500,000 pieces in 2007 (see also Rhyne et al.^5^, Dee et al.^3^). International trade in live corals further increased in 2016–2018^13^. Prior to 2018, Indonesia and Fiji were the largest exporters of hard (order Scleractinia) corals (i.e., accounting for 70.0% and 10.3% of global exports, respectively^1^), but both these countries imposed significant constraints on wild coral harvesting (in 2017–2018) due to concerns of overharvesting, which significantly changed the nature of global coral trade.

Environmental concerns regarding coral harvest fisheries have mostly focused on the potential for localized depletion of highly vulnerable or heavily targeted coral species^4,14^. Many of the most heavily targeted coral species have large fleshy polyps (often referred to as Large Polyp Stony corals; LPS corals), which are presumed to be slow growing and long lived^14^. Despite a lack of relevant biological information for many of these species, it is presumed that such species will be highly vulnerable to over-fishing^2,14^. Particular concern also exists where fisheries exploitation is compounded by rapid and accelerating environmental change^14^.

In Australia, commercial coral harvest fisheries are managed at the state/territory level with major fisheries in Queensland (Queensland Coral Fishery; QCF), Western Australia (Western Australian Marine Aquarium Fish Managed Fishery; WAMAFMF), and the Northern Territory (Northern Territory Aquarium Fishery; NTAF). The QCF operates over a total area of 24,000 km^2^ in permitted zones of the Great Barrier Reef Marine Park (GBRMP). The QCF has the largest annual Total Allowable Commercial Catch (TACC) of all Australian coral fisheries at 200 t, which is split between 60 t of ‘specialty’ or LPS corals and 140 t of ‘other’ corals; which includes branching taxa such as the Acroporidae^15^. Concerns have previously been raised regarding the potential for the localised depletion of specific coral species in areas of concentrated fishing activity^16^, combined with the threat posed by extrinsic disturbances such as cyclones, outbreaks of crown-of-thorns starfish and mass coral bleaching^15,17,18^. The WAMAFMF is the second largest coral fishery. It is a low volume and high value fishery that operates over a total gazetted area of 20,781 km^2^ along Australia’s west coast, with a TACC of 15 t for hard and soft corals. The NTAF operates in all inland, estuarine, and marine waters to the outer boundary of the Australian Fishing Zone (AFZ) in NT waters, incorporating an area of 523,946 km^2^ of marine habitat. This fishery has the only taxon-specific quota levels, typically assigning 80 kg for individual species, and 160 kg for species groups.

A recent re-assessment of the QCF^19^, as part of the necessary process of seeking Wildlife Trade Operation (WTO) approval from CITES, has highlighted the growth in this fishery and the continued prominence of LPS corals since the last comprehensive assessments of global import and export trends in the trade of ornamental corals^1,5^. This re-assessment examined QCF catch trends from the 2006/2007 to 2019/2020 financial years, with a similar re-assessment now underway for the WAMAFMF and NTAF as WTO approval expires in late 2022. Importantly, while *Acropora* corals form a major and increasingly large proportion of the QCF coral harvest^19^, LPS species remain an important component both in volume and economic contribution^1^. The three major LPS coral families (Lobophylliidae, Merulinidae, Euphylliidae) together accounted for ~ 41% of the QCF’s total catch composition in the 2019–2020 financial year. Furthermore, there are 6 species that are of particular economic, biological, and ecological interest, namely *Homophyllia* cf. *australis* (Milne Edwards & Haime, 1848; Lobophyllia), *Micromussa lordhowensis* (Veron & Pichon, 1982; Lobophylliidae), *Catalaphyllia jardinei* (Saville-Kent, 1893; Merulinidae), *Trachyphyllia geoffroyi* (Audouin, 1826; Merulinidae), *Euphyllia glabrescens* (Chamisso & Eysenhardt, 1821; Euphyllidae), and *Duncanopsammia axifuga* (Milne Edwards & Haime, 1848; Dendrophylliidae). Together, these species accounted for over 35% of 2019–2020 total catch composition for the QCF, representing a total of 14.2 t or 317,718 coral pieces^19^. The Ecological Risk Assessment category for some of these heavily targeted LPS corals has recently been upgraded in the Queensland Coral Fishery, with *H.* cf. *australis* and *M. lordhowensis* now considered to be at “extreme” risk of experiencing an “undesirable event” as a result of fishery actions, while *E. glabrescens* and *T. geoffroyi* were upgraded to the “high risk” category^20^.

There are uncertainties regarding the sustainability of harvest levels and harvest limits for Australian coral harvest fisheries^19^, mainly because the status and trends for targeted coral species and stocks are largely unknown. Harvest logs for these fisheries are currently reported in terms of weight, however, it can be difficult to understand the ecological relevance of recorded catch from this metric alone. Fundamentally, most existing accessible data on the abundance of harvested coral species based on either coral cover or colony densities (e.g., those outlined in Mellin et al.^21^) are not useful for establishing the ecological context of weight-based harvest limits. Additionally, some corals are collected as fragments as opposed to entire individuals, causing further difficulties if attempting to utilise coral cover or abundance data for fisheries monitoring. Instead, estimates of harvestable or ‘standing’ biomass would provide a better unit of quantification for assessment of coral harvest quotas and the ecological impact of harvesting^22^. To bridge this gap between harvest and ecological impact, Longenecker et al.^23^ utilised size-weight relationships to estimate standing biomass of *Acropora* corals, suggesting that establishing these relationships in other corals is likely to be a viable approach that can provide managers with a relatively simple methodology able to place fisheries harvests into an ecological context (see also Pacey et al.^24^).

To facilitate improved management of Australia’s coral harvest fisheries (and perhaps internationally), this study modelled the relationship between maximum diameter and coral weight for six key LPS coral species: *Catalaphyllia jardinei*, *Duncanopsammia axifuga*, *Euphyllia glabrescens*, *Homophyllia* cf. *australis*, *Micromussa lordhowensis*, and *Trachyphyllia geoffroyi*. Aside from providing a mechanism to calculate standing biomass of these coral species, establishing size-weight relationships for these corals will provide an opportunity to assess previously unexamined biological characteristics of these species relating to these described relationships, such as whether these corals exhibit isometric or allometric growth patterns. Moreover, size-weight information is combined with in situ video transects to demonstrate the utility of this method for estimating standing biomass of individual corals.

## Results

### Size and weight trends

A total of 2548 corals were measured across the six study species: *Catalaphyllia jardinei* (n = 43), *Duncanopsammia axifuga* (n = 219), *Euphyllia glabrescens* (n = 265), *Homophyllia* cf. *australis* (n = 436), *Micromussa lordhowensis* (n = 685), *Trachyphyllia geoffroyi* (n = 900). The majority of coral pieces were collected in Queensland (1,986 corals; ~ 78%), with ~ 21% (536) corals collected in Western Australia and ~ 1% (26) corals sampled in the Northern Territory. Due to uncertainties in taxonomy and range for the corals *Homophyllia* cf. *australis* and *Micromussa lordhowensis*, only corals from QLD were used to model data (Table 1).

**Table 1:** Coral pieces used to construct size-weight models showing the state that the samples were obtained from, number (n), mean ± *SE*, minimum (min) and maximum (max) value of samples by (a) coral weight (g), and (b) coral diameter (cm) for each species (*Catalaphyllia jardinei*, *Duncanopsammia axifuga*, *Euphyllia glabrescens*, *Homophyllia* cf. *australis*, *Micromussa lordhowensis*, *Trachyphyllia geoffroyi*).

Species	State	n	Mean ± *SE*	Min	Max
(a) Weight (g)					
*Catalaphyllia jardinei*	QLD	43	166.19 ± 33.97	17	985
*Duncanopsammia axifuga*	WA	172	258.28 ± 25.55	10	2750
	QLD	47	126.06 ± 15.05	17	483
Euphyllia glabrescens*	WA	191	202.02 ± 18.67	6	1530
	QLD	48	130.87 ± 22.47	16	948
	NT	26	24.96 ± 2.48	5	53
*Homophyllia* cf. *australis*	QLD	436	72.88 ± 2.12	8	448
*Micromussa lordhowensis*	QLD	685	314.06 ± 11.26	10	1952
*Trachyphyllia geoffroyi*	QLD	727	249.95 ± 7.85	8	1695
	WA	173	93.60 ± 5.76	8	1695
(b) Diameter (cm)					
*Catalaphyllia jardinei*	QLD	43	7.52 ± 0.46	3.90	13.40
*Duncanopsammia axifuga*	WA	172	11.20 ± 0.37	2.60	30.50
	QLD	47	10.27 ± 0.54	4.50	20.70
Euphyllia glabrescens*	WA	191	8.32 ± 0.25	2.10	19.20
	QLD	48	7.67 ± 0.46	3.20	17.60
	NT	26	4.28 ± 0.22	2.00	6.30
*Homophyllia* cf. *australis*	QLD	436	5.76 ± 0.05	2.50	9.75
*Micromussa lordhowensis*	QLD	685	11.48 ± 0.16	3.44	25.60
*Trachyphyllia geoffroyi*	QLD	727	9.20 ± 0.08	2.80	19.29
	WA	173	6.96 ± 0.10	3.70	10.60

*Some evidence to suggest *E. glabrescens* may represent different species across states (based on tentacle morphology, tank compatibility, planulation time, and size).

Australian coral fisheries tend to harvest small pieces or colonies of LPS corals, as shown by the mean diameter and weight of corals provided for the current study. *M. lordhowensis* corals had the highest average weight and diameter, followed by *D. axifuga*, *T. geoffroyi*, *E. glabrescens*, *C. jardinei*, with *H.* cf. *australis* having the lowest average weight (Table 1). There was substantial statistical evidence to suggest that all coral species differed in weight (Bayesian ANOVA pairwise comparison, P < 0.01), with the exception of *C. jardinei* and *E. glabrescens* (Bayesian ANOVA pairwise comparison, P = 0.341). Similarly, there was strong evidence to suggest a difference in average diameter between all corals (Bayesian ANOVA pairwise comparison, P < 0.01), again with the exception of *C. jardinei* and *E. glabrescens* (Bayesian ANOVA pairwise comparison, P = 0.308). For corals which were sampled from multiple states (*D. axifuga*, *E glabrescens*, *T. geoffroyi*), there was significant evidence (Bayesian ANOVA pairwise comparison, P < 0.01) to suggest that all samples differed between states in terms of weight. Coral weight samples from QLD were significantly lower than those from WA for *D. axifuga*. For *E. glabrescens*, weight (g) of samples from the NT were lowest, followed by QLD, with WA being on average higher, all states being significantly different to each other. For *T. geofroyi*, corals sampled from QLD had significantly higher weight than those from Western Australia. For maximum diameter (cm), samples from the NT were significantly lower than samples from both QLD and WA, but QLD and WA were also statistically different. For *T. geffroyi*, the maximum diameter of samples from QLD was higher than samples collected from Western Australia.

### Size–weight relationships

**Figure 1:**
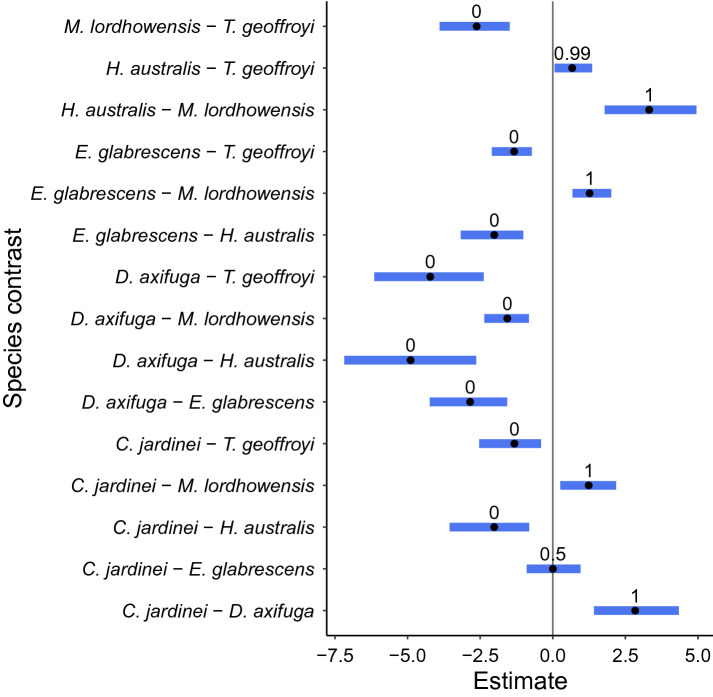
By species (*Catalaphyllia jardinei*, *Duncanopsammia axifuga*, *Euphyllia glabrescens*, *Homophyllia* cf. *australis*, *Micromussa lordhowensis*, *Trachyphyllia geoffroyi*) pairwise comparison of species coefficient estimate on the non-linear ‘*c*’ parameter, where blue bars represent 95% C.I. (credible interval), black point represents estimate of effect, and number above blue bar represents the probability that P > 1, where terms outside the 5–95% interval indicate statistical significance.

The relationship between size and weight for all individual species was well represented by a normally distributed non-linear power formula, which outperformed the alternative linear and exponential functions (LOO, closest elpd_diff = − 348.8, se_diff = 19.7 for the linear alternative). However, the relationship between size and weight was statistically different among coral species (Pairwise comparison on ‘c’ parameter for ANCOVA type model, 95% HDP interval, median point estimate) with the exception of *C. jardinei* and *E. glabrescens*, which were the only two species which did not significantly differ in the ‘*c*’ parameter species coefficient (P > 1 = 0.50; Fig. 1). All species therefore exhibit fundamental differences in their size to weight relationship over the measured diameter ranges. The contrast between *Homophyllia* cf. *australis* and *Trachyphyllia geoffroyi* was the only other contrast that did not receive a 0 or 1 probability; however, their posterior probability value is still considered highly significant (P > 1 = 0.99). From a fisheries perspective, this means that all species should be assessed and considered separately in terms of their size-weight relationship to properly estimate biomass and place the fisheries impact of each species into an appropriate, relevant, ecological context. Following this finding, the decision was made to model all species separately using an intercept only ‘*a*’ and ‘*b*’ parameter approach.

**Figure 2:**
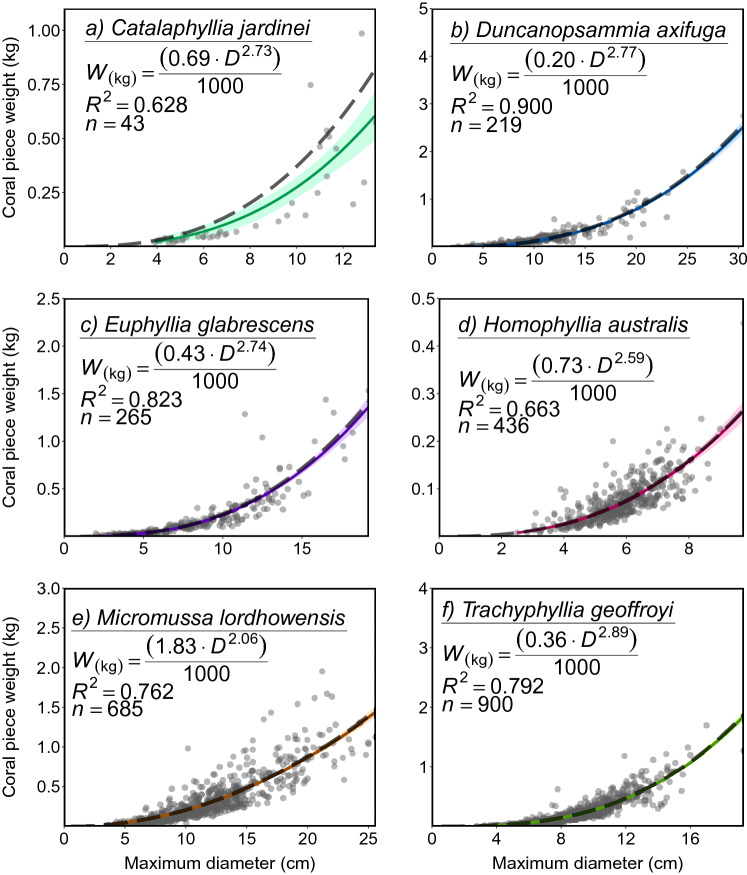
Modelled relationship between maximum diameter (cm) and coral weight (g) for the species: (**a**) *Catalaphyllia jardinei*, (**b**) *Duncanopsammia axifuga*, (**c**) *Euphyllia glabrescens*, (**d**) *Homophyllia* cf. *australis*, (**e**) *Micromussa lordhowensis*, (**f**) *Trachyphyllia geoffroyi*. Probability band of the model (0.95) is indicated by the coloured bands surrounding model line, while grey dots indicate individual datapoints, and dashed grey model line represents the displayed approximated power relationship equation. For a full list of non-linear parameter constant estimates, SE, and High Density Intervals see Online Appendix A.

The relationship between coral diameter and weight as modelled in the current instance was able to account for the majority of variation in data across all species, with *R*^*2*^ values ranging from 0.63 (*C. jardinei*) to 0.90 (*D. axifuga*) (Fig. 2). The size-weight equation (broken grey line) was well estimated using the intercept only parameter approach, following the statistical model line (coloured line) and staying within the 95% confidence band in most instances, with the exception of *C. jardinei*. In the instance of the model line and the estimated equation, both appear to represent somewhat of an underestimate. This reflects greater error on the ‘*a*’ and ‘*b*’ non-linear intercept parameters, most likely resulting from sample size limitations.

Model and equation lines typically bisect the major cluster of samples (e.g., *H.* cf. *australis*, *M. micromussa*), although there is evidence of underestimation in the higher maximum diameter range for some species (e.g., *M. micromussa*, *T. geoffroyi*). By all indications in the current study, all selected coral species with the exception of *C. jardinei* (2.73 ± 0.35 S.E., 95% CI 2.14–3.18, P < 3 = 0.22) appear to exhibit allometric growth, with exponents for all species falling below ~ 3 (P < 3 = 1) (Fig. 2).

### Biomass per unit area estimation

Across all 204 transects conducted in both Queensland and Western Australia, *Catalaphyllia jardinei* had the highest average biomass per unit area (g·m^−2^), followed by *Duncanopsammia axifuga*, *Euphyllia glabrescens*, *Micromussa lordhowensis*, *Trachyphyllia geoffroyi*, and *Homophyllia australis* (Table 2). As expected, when considering the described exponential size-weight relationship, larger corals contribute disproportionately to coral transect biomass per unit area. This is indicated by the considerable deviation between the sample and population means for *C. jardinei* and *D. axifuga* as shown by standard error values and comparison of mean to median values (Table 2). *C. jardinei* in particular had some relatively extreme estimates, with a maximum biomass per unit area value of 33,745.09 g·m^2^ for one transect resulting from a concentration of large specimens (18 corals > 50 cm), with one coral having a maximum diameter of 90.67 cm.

**Table 2:** The (a) average transect biomass per unit area (g m^2^) and (b) maximum diameter (cm) of corals recorded on transects, including state that the sample was obtained from, number of transects (n), mean ± *SE*, median, minimum (min) and maximum (max) value, by species.

Species	State	n	Mean ± *SE*	Median	Min	Max
(a) Biomass (g·m^2^)						
*Catalaphyllia jardinei*	QLD	16	6406.00 ± 2985.86	38.00	0.23	33,745.09
*Duncanopsammia axifuga*	WA	31	102.29 ± 50.45	8.87	0.26	1462.80
	QLD	2	5.48 ± 5.10	5.48	0.38	10.58
*Euphyllia glabrescens*	WA	27	17.44 ± 8.08	5.63	0.18	216.62
	QLD	18	60.05 ± 27.69	3.87	0.01	471.65
*Homphyllia* cf. *australis*	QLD	45	6.01 ± 1.38	3.23	0.27	49.28
*Micromussa lordhowensis*	QLD	18	10.20 ± 2.61	7.28	0.48	46.58
*Trachyphyllia geoffroyi*	WA	29	4.31 ± 0.84	2.21	0.10	20.00
	QLD	18	16.28 ± 2.40	4.76	1.69	37.53
(b) Diameter (cm)						
*Catalaphyllia jardinei*	QLD	16	14.10 ± 2.76	8.25	2.79	40.60
*Duncanopsammia axifuga*	WA	31	10.20 ± 1.01	8.28	4.29	27.70
	QLD	2	8.94 ± 3.77	8.94	5.17	12.70
*Euphyllia glabrescens*	WA	27	11.70 ± 1.56	10.2	1.00	24.80
	QLD	18	6.37 ± 0.54	5.72	3.02	15.40
*Homphyllia* cf. *australis*	QLD	45	4.12 ± 0.17	3.80	2.52	7.01
*Micromussa lordhowensis*	QLD	18	9.29 ± 1.00	8.08	3.50	20.00
*Trachyphyllia geoffroyi*	WA	29	5.49 ± 0.38	5.04	3.76	10.10
	QLD	18	5.04 ± 0.23	5.14	1.64	7.41

For species where a sufficient number of transects were available for between-state comparison of biomass (i.e., *Euphyllia glabrecens* and *Trachyphyllia geoffroyi*), only *T. geoffroyi* was found to be significantly different (Bayesian ANOVA pairwise comparison, P = 1), with transects from Western Australia on average containing significantly lower biomass per unit area. Similarly, the average diameter of *E. glabrescens* was found to be significantly different (P = 1) between regions, with a smaller average size observed for samples from Queensland.

## Discussion

Establishing size-weight relationships for heavily targeted coral species is an important first step towards informing sustainable harvest limits^19^. Placing coral harvests into an ecological context is a core requirement for implementing a defensible stock assessment strategy, and this need is particularly critical given escalating disturbances and widespread reports of coral loss^7,17,25^. Using these relationships, managers can now easily sample and calculate biomass per unit area. It is important to point out that all sites sampled in our study represent fished locations, and there is no information available to test whether standing biomass has declined due to sustained coral harvesting at these locations. While these data may now provide a critical baseline for assessing the future effects of ongoing fishing, it is also important to sample at comparable locations where fishing is not permitted or has not occurred (where possible), to test for potential effects of recent and historical harvesting.

Biomass per unit area data presented herein highlights the highly patchy abundance and biomass of targeted coral species^14^, which is evident based on the often vastly different mean and median values (Table 2). Examining biomass per unit area estimates for *C. jardinei* for example, which returned some of the highest biomass estimates, the 33.75 kg·m^−2^ maximum estimate from a transect stands as an extreme outlier, with 12 of the 16 other transects being below 0.2 kg·m^−2^. This indicates the challenges of managing species that occur in patchily distributed concentrations, particularly in a management area the size of the QCF. It is also important to note, these estimates are generated only on transects where the target species occurred, and therefore, should technically not be considered as an overall estimate of standing biomass. While the estimation of size-weight relationships is a step towards a standing biomass estimate, many challenges remain in terms of sampling or reliably predicting the occurrence of these patchily distributed species. Bruckner et al.^14^ attempted to overcome this management challenge in a major coral fishery region of Indonesia by categorising and sampling corals (in terms of coral numbers) in defined habitat types, and then extrapolating to estimated habitat area based on visual surveys and available data. This approach, utilising size-weight relationship derived biomass per unit area estimates (instead of coral numbers), may be a viable method for the QCF, however much more information is needed to understand the habitat associations (e.g., nearshore to offshore), and environmental gradients that influence the size and abundance of individual corals. Fundamentally, it is also clear that much more data is required to effectively assess the standing biomass of aquarium corals in the very large area of operation available to Australian coral fisheries.

These corals are found in a range of environments, and it is important to consider available information on life history if attempting to use coral size-weight relationships to inform management strategies via standing biomass estimation. All corals in this study can be found as free living corals (at least post-settlement) in soft-sediment, inter-reefal habitats, from which they are typically harvested by commercial collectors^19^. However, only four of the 6 species are colonial (*C. jardinei, D. axifuga, E. glabrescens, M. lordhowensis*) while the remaining two species (*H.* cf. *australis* and *T. geoffroyi*) are more typically monostomatous or solitary. As indicated in previous work^24^, if larger colonial corals were to be fragmented during harvesting instead of removed entirely, fishery impacts would likely be lessened^24^. Given the power relationship between coral maximum diameter and weight, larger corals contribute disproportionately to the total available biomass of each species in a given area. The potential environmental benefit of leaving larger colonies (at least partially) intact is not limited to impacts on standing biomass, as this practice would likely be demographically beneficial given the greater reproductive potential (i.e., fecundity) of larger colonies, which also do not need to overcome barriers to replenishment of populations associated with new recruits (i.e., high mortality during and post-settlement^26^). This conclusion was drawn largely from data on branching taxa (e.g., *Acropora*), which are relatively resilient to fragmentation and commonly undergo fragmentation as a result of natural processes^27–29^. *D. axifuga* can be considered to exhibit a relatively similar branching growth form, however, the growth form of *E. glabrescens* and *C. jardine*i changes with size, moving from small discrete polyps to large phaceloid and flabello-meandroid colonies, respectively^19^. While larger colonies of *E. glabrescens* and *C. jardinei* may be relatively resilient to harvesting via fragmentation, the same may not be true for smaller colonies, or species with massive growth forms such as *M. lordhowensis*. Typically, for each species, the average reported weight was quite low, coinciding with the lower end of the sampled maximum diameter range. For colonial species, the harvested smaller maximum diameters (if fragments) are ideal from an ecological perspective as this will have the least impact possible on standing biomass, and may also leave a potentially mature breeding colony intact. Ultimately, in light of these considerations, the development of uniform and standardised industry-wide harvest guidelines to balance economic and ecological outcomes may be necessary. The development of these guidelines would require consultation with commercial harvesters, as well as considerable additional work in measuring ecological impacts and better understanding the cost of these impacts from an economic perspective. Conversely, if whole colonies are collected, which is necessarily the case for solitary species such as *H.* cf. *australis* and *T. geoffroyi* (and potentially smaller colonies of other species such as *E. glabrescens* and *C. jardinei*); smaller colonies may be collected before they reach sexual maturity, hindering their ability to contribute to population replenishment. Therefore, collection of small fragments should be encouraged for colonial species; while for monostomatous species where this is not possible, introduction of a minimum harvest size based on sexual maturity should be considered.

Additionally, the need for further consideration of the selectivity of ornamental coral harvest fisheries^3,4,30^ when assessing standing biomass is evident. Due to various desirable traits, the majority of available biomass may not be targeted by collectors. As emphasised in this study, the focus on smaller corals is indicative of the trend towards collection of most of these species at the lower portion of their size range, at least compared to some of the maximum sizes recorded on transects (e.g., see Tables 1 and 2, section b). However, it is also important to consider that transects were conducted in areas subject to commercial collection and are likely to skew results and prevent clear conclusions relating to size selectivity. Sampling of unfished populations (i.e., any residing outside of permitted fishing zones) and/or spatial and temporal matching of catch data and transect data across a larger sample of operators will be required to properly address industry size selectivity trends. For instance, only 17.5% of *C. jardinei* corals measured on transects fell within the diameter range represented by data obtained from collectors, with 81.9% of corals measured on transects exceeding this range. If it is viable to collect fragments from larger colonies (which does appear to be the case for some corals such as *C. jardinei*), then a larger proportion of standing biomass outside of this size range could be targeted by fishers. As an additional consideration, only desirable colour morphs of these corals will be harvested, and due to lack of appropriate data, the prevalence of these morphs remains unclear. *H.* cf. *australis* and *M. lordhowensis* for example often occur in brown colour morphs, which are far less popular in markets where certain aesthetic qualities (e.g., specific, eye-catching colours or combinations of colours) are desired, such as the ornamental aquarium industry. Even without delving into further considerations such as heritability of phenotypic traits, management conclusions drawn from standing biomass estimates may be ineffective in the absence of efforts to account for selectivity in this fishery.

The relationship between size and weight was found to differ between all corals, with the exception of *C. jardinei* and *E. glabrescens*. There can be some moderate similarity in skeletal structure between these two species, particularly between small colonies, reflecting the similar maximum diameter range of sampling in the current study. Subsequently, inherent physiological constraints may be imposed on corals that prevent the maintenance of growth rates between corals of smaller and larger sizes, for example, as the surface area to volume ratio declines with growth^31^. In the current study, all corals, with the exception of *C. jardinei*, showed evidence of allometric growth, as exhibited by an estimated exponent value different to 3. Sample size for *C. jardinei* was greatly limited, as this species typically forms extensive beds, and are rarely brought to facilities as whole colonies. Therefore, the lack of evidence for allometric growth may reflect higher error for the species coefficient parameter due to the comparatively small sample size for this species. This suggests that mass would not increase consistently with changes in colony size in 3 dimensions^31^, which seems likely considering the change in exhibited form described for *E. glabrescens* and *C. jardinei* previously. In the current context, this indicates that the estimated ‘*a*’ and ‘*b*’ constants are likely to vary as the sample range increases, reflecting the changes in the size-weight relationship between smaller and larger samples of these species. Therefore, ideally, these models should incorporate data that reflect the maximum diameter range of the species in the region of application to allow increased accuracy of biomass estimation. To achieve this will require additional fishery-independent sampling, as large colonies are rarely collected whole, though may be collected as fragments depending on the species. Sampling may be challenging for some species given the difficulty of physically collecting and replacing large whole colonies, particularly for inter-reefal species such as *M. lordhowensis*, which can occur in deep, soft sediment habitat, subject to strong currents. Importantly, obtaining ex situ or in situ growth rate data should be considered a priority for the management of heavily targeted species. This data is likely to be another necessary component (in conjunction with size-weight relationships) of any stock assessment model developed for LPS corals, and may also eliminate the need to collect large sample colonies to improve estimated size-weight relationships.

The disproportionate focus on smaller corals (i.e., corals in the current study averaged between 4.28 and 11.48 cm in maximum diameter) is likely to lead to an underestimation of weight in corals at greater diameters when used as inputs for size-weight models. This may explain the apparent minor underestimation observed in some species (e.g., *M. micromussa*, *T. geoffroyi*). In the current context, this represents an added level of conservatism with estimates obtained from these equations. While the relationship between size and weight was particularly strong for some species, (mainly *D. axifuga* and *T. geoffroyi*), for other species, such as *M. lordhowensis*, growth curves tended towards underestimation at larger diameter values. As the mass of a coral is reflective of the amount of carbonate skeleton that has been deposited^32^, the coral skeleton may increase disproportionately to coral diameter if or when corals start growing vertically. For example, in massive corals such as *M. lordhowensis*, vertical growth (i.e., skeletal thickening) is often very negligible among smaller colonies, with thickening of the coral skeleton only becoming apparent once the coral has reached a threshold size in terms of horizontal planar area. Additional fisheries-independent sampling outside of the relatively narrow size range of harvested colonies will be required to address this source of error in future applications. Ecological context in the form of fishery independent data on stock size and structure is essential for effective management, especially in ensuring that exploitation levels are sustainable and appropriate limits are in place. Coral harvest fisheries offer managers an ecologically and biologically unique challenge, as the implementation of standard fisheries management techniques and frameworks is hampered by their coloniality and unique biology, as well as a general lack of relevant data for assessing standing biomass and population turnover, not to mention the evolving taxonomy of scleractinian corals^33^. Similarly, fishery-related management challenges such as the extreme selectivity in terms of targeted size-ranges and colour-morphs, plus the potentially vast difference in the impact of various collection strategies (i.e., whole colony collection vs fragmentation during collection) also complicates the application of typical fisheries stock assessment frameworks. The relationships and equations established in the current work offer an important first step for coral fisheries globally by laying the groundwork for a defensible, ecologically sound management strategy through estimation of standing biomass, thus bridging the gap between weight-based quotas and potential environmental impacts of ongoing harvesting. It is important to note that the species selected for the current work do not represent the extent of heavily targeted LPS corals. For example, *Fimbriaphyllia ancora* (Veron & Pichon, 1980), *Fimbriaphyllia paraancora* (Veron, 1990), *Cycloseris cyclolites* (Lamark, 1815), and *Acanthophyllia deshayesiana* (Michelin, 1850) are examples of other heavily targeted corals of potential environmental concern^19^, and management would also benefit from the estimation of size-weight relationships for these species. Moving forward, the next challenge for the coral harvest fisheries will be to comprehensively document and track the standing biomass of heavily targeted and highly vulnerable coral stocks, explicitly accounting for fisheries effects and also non-fisheries threats, especially global climate change.

## Methods

### Data collection

**Figure 3:**
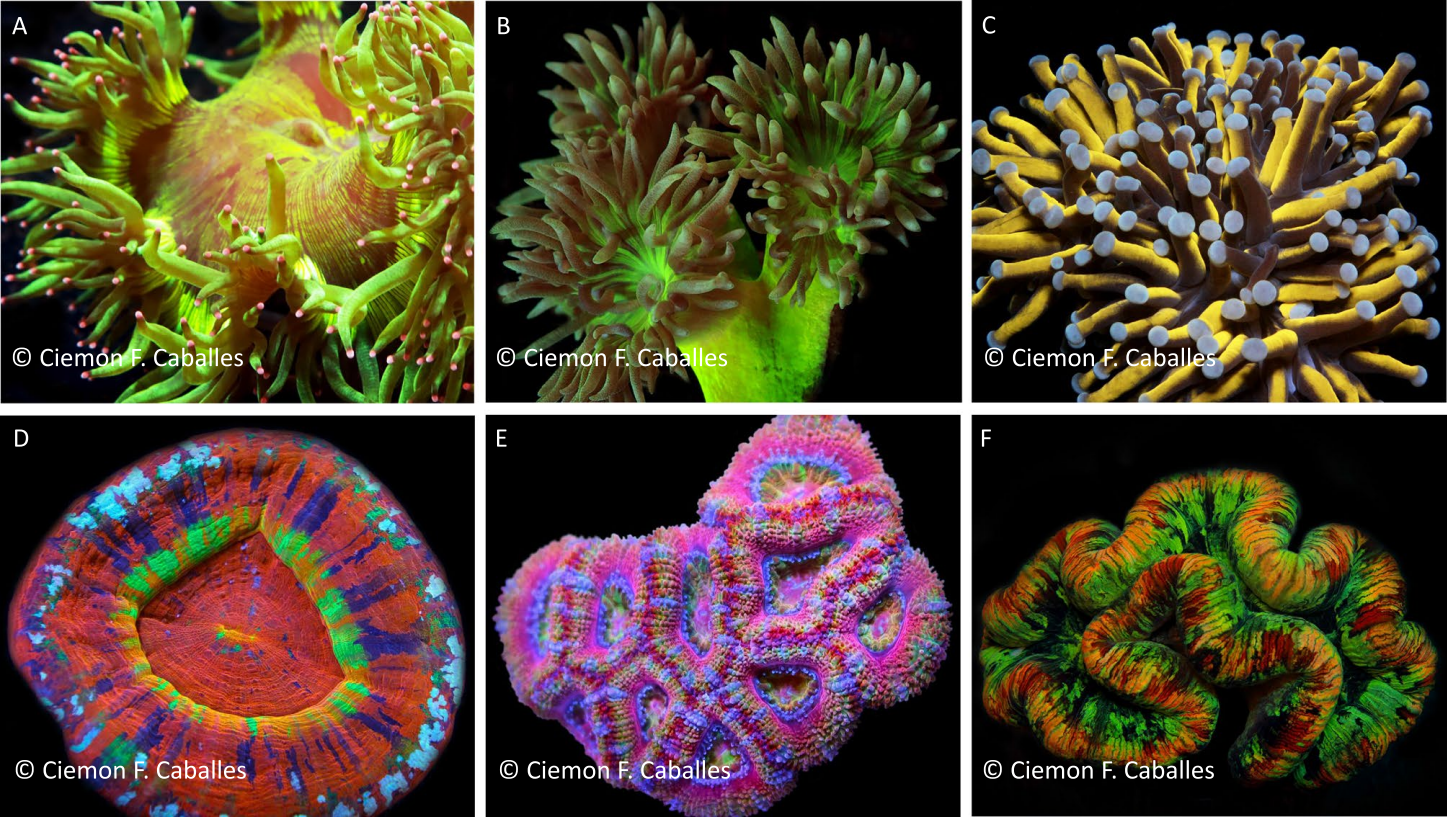
LPS (Large Polyp Stony) corals targeted for commercial collection used in the current study, namely; (**A**) *Catalaphyllia jardinei,* (**B**) *Duncanopsammia axifuga* (**C**) *Euphyllia glabrescens* (**D**) *Homophyllia* cf. *australis*, (**E**) *Micromussa lordhowensis*, (**F**) *Trachyphyllia geoffroyi*.

The size-weight relationships of six coral species (*Catalaphyllia jardinei*, *Duncanopsammia axifuga*, *Euphyllia glabrescens*, *Homophyllia* cf. *australis*, *Micromussa lordhowensis*, and *Trachyphyllia geoffroyi*; see Fig. 3) was investigated using coral pieces provided by commercial collectors between March 2016 and July 2020. Samples were collected from Queensland, Western Australia, and the Northern Territory. Collection is typically far more geographically focused in the latter two states, and so locations were supposed to represent the major areas of operation in each state. Species selected for this study were prioritised based on their importance to coral fisheries exports across Western Australia, Northern Territory, and Queensland; and the perceived risk to these species in terms of overfishing and/or fishery independent threats (as identified in Pratchett et al.^15^).

Data used to generate size-weight relationship models for each species was collected in collaboration with fishery operators. The maximum diameter and perpendicular diameter of each coral was recorded to the nearest millimetre using callipers, or a ruler for larger corals, while weight was recorded to the nearest gram using an electronic scale. Corals were left to drain freely for 2–5 min prior to processing. Samples were mostly intact whole coral colonies (25 fragments total, < 0.01% of total sample size), with excess substrate removed prior to weighing. Care was taken to also remove excess water prior to weighing of corals. In addition to size and weight measurements, site, region, and state from which the sample was obtained was also recorded.

To demonstrate the utility of modelled size-weight relationships, we estimated the standing biomass of the six focal species (*Catalaphyllia jardinei*, *Duncanopsammia axifuga*, *Euphyllia glabrescens*, *Homophyllia* cf. *australis*, *Micromussa lordhowensis*, and *Trachyphyllia geoffroyi*) across a total of 204 video transects conducted in two states (QLD, WA) across six locations (Cairns, Dampier, Exmouth, Karratha, Mackay, Southern Great Barrier Reef), 15 reefs, and 34 sites. Video transect surveys were conducted on SCUBA using a 50-m transect tape deployed along a depth contour ranging from 0 to 20.9 m. A GoPro Hero 7 mounted on a camera jig was used to record the substrate along each depth contour. The camera jig was made using 1.2. × 1.0 m PVC conduit pipes, with the camera mounted on the 1.2 m pipe perpendicular to the 1 m piece in a ‘T’ configuration. The end of the 1 m pipe was placed on, or as close as practicable to the transect line and/or coral while swimming from one end of the transect line to the other (giving recorded belt transects a dimension of 50 × 1 m). Care was taken to maintain camera angle, as well as avoid damage to corals and other benthic biota during surveys.

### Processing and analyses

A Bayesian non-linear regression approach was used to model the relationship between maximum coral colony diameter in cm (*D*) and weight in g (*W*) within a gaussian distribution (see Eq. 1). In this approach, the non-linear predictor (‘$\eta$’) for the primary parameter ‘$\mu$’ can be described following Eq. (2), with the covariate (‘*D’*, i.e., maximum diameter in cm) and the nonlinear parameters ‘*a*’ and ‘*b*’, each of which represent an intercept-only predictor parameter and are therefore calculated as constants. The term ‘*f’* defines the structure of the user supplied function (Eq. 3), which in this case is a two-factor power function. Size-weight relationships have been well described by power functions in corals previously^23,24^, where coral weight (in the current case defined as ‘*W*’, g) is equal to a constant scaling factor (‘*a*’) multiplied by the corresponding coral diameter (‘*D*’, cm), to the power of a constant exponent (‘*b*’). For further explanation of the distributional non-linear modelling approach utilised in this study, see Bürkner^34^.

1 $${W}_{i} \sim N({\mu }_{i}, {\sigma }_{i})$$

2 $${\eta }_{\mu }= f(D, a, b)$$

3 $$f= a{D}^{b}$$

Bayesian statistical inference was performed via the Hamiltonian Monte Carlo algorithm and its extension No-U-Turn Sampler (NUTS) using the statistical software Stan to model non-linear relationships, estimate the parameters of the user supplied power function, and investigate biomass estimation results. This was accomplished using the ‘brm’ function within the R^35^ (ver 4.1.3) package ‘brms’^36^. Efficient approximate leave-one-out (LOO) cross validation for Bayesian models via Pareto smoothed importance sampling (PSIS)^37^ was conducted to establish the validity of the non-linear power size-weight model and its predictive capacity using the function ‘loo’ within the package ‘brms’.

Alternative models (e.g., linear, non-linear exponential) were investigated visually using the ‘geom_smooth’ function (method loess) in the package ggplot2^38^ with decisions validated using the ‘loo’ function. Previous work describing size-weight relationships in corals^15^ was used to inform the initial values of set priors, with further modification of these priors applied where necessary during model validation. A Bayesian ANOVA (utilising median values) and pairwise comparisons were used to assess statistical difference in weight (g) and biomass per unit area (g m^2^) and maximum diameter (cm) between species. To further compare the size-weight relationship between species, a lognormally distributed ANCOVA-type model was used. A pairwise comparison approach was utilised to compare the differences at the ‘*c*’ non-linear parameter, with further investigation of one-sided posterior probability conducted using the package ‘emmeans’. For a full list of priors see Online Appendix B.

To measure the size of each target coral recorded during video transect surveys, video frames were captured and analysed using the ‘set scale’ and ‘measure’ tools in the software ImageJ^39^. The camera jig was used to set the scale of captured frames for measurement when the 1-m conduit was over or directly next to the target coral colony. A maximum colony length and colony width was then recorded for each coral. The biomass in grams of each coral was then estimated via the relevant species-specific size-weight relationship model equation derived from the estimated parameters of the user supplied function (Eq. 2). To convert transect biomass estimate data into a biomass per unit area estimate, biomass for each growth form group was averaged to the transect level and divided by total transect area (50 m × 1 m).

## Supplementary Information

Supplementary Information.

## Data Availability

Restrictions apply to the availability of these data as it contains information obtained from third party sources (i.e., fishery operators) considered to be of a commercially sensitive nature, and so is not publicly available.

## References

[1] Wood, E., Malsch, K. & Miller, J. International trade in hard corals: review of management, sustainability and trends. in *Proc. 12th ICRS Cairns, Aus, 9–13 July 2012* (2012).

[2] Rhyne AL, Tlusty MF, Kaufman L (2014). Is sustainable exploitation of coral reefs possible? A view from the standpoint of the marine aquarium trade. Curr. Opin. Environ. Sustain..

[3] Dee LE, Horii SS, Thornhill DJ (2014). Conservation and management of ornamental coral reef wildlife: Successes, shortcomings, and future directions. Biol. Conserv..

[4] Harriott, V. J. *The Sustainability of Queensland’s Coral Harvest Fishery*. (CRC Reef Research Centre, 2001).

[5] Rhyne AL, Tlusty MF, Kaufman L (2012). Long-term trends of coral imports into the United States indicate future opportunities for ecosystem and societal benefits. Conserv. Lett..

[6] Bruno JF, Valdivia A (2016). Coral reef degradation is not correlated with local human population density. Sci. Rep..

[7] Hughes TP (2017). Global warming and recurrent mass bleaching of corals. Nature.

[8] Fabricius KE (2005). Effects of terrestrial runoff on the ecology of corals and coral reefs: Review and synthesis. Mar. Pollut. Bull..

[9] Wear SL (2016). Missing the boat: Critical threats to coral reefs are neglected at global scale. Mar. Policy.

[10] Burke, L., Reytar, K., Spalding, M. & Perry, A. *Reefs at risk revisited*. (World Resources Institute, 2011).

[11] Hughes TP (1994). Catastrophes, phase shifts, and large-scale degradation of a Caribbean coral reef. Science.

[12] Negri AP, Smith LD, Webster NS, Heyward AJ (2002). Understanding ship-grounding impacts on a coral reef: Potential effects of anti-foulant paint contamination on coral recruitment. Mar. Pollut. Bull..

[13] Food and Agriculture Organization of the United Nations & Pavitt, A. , Malsch, K. , King, E. , Chevalier, A. , Kachelriess, D. , Vannuccini, S. , Friedman K. *CITES and the sea: Trade in commercially exploited CITES-listed marine species*. (Food & Agriculture Org., 2021).

[14] Bruckner, A. W. & Borneman, E. H. Developing a sustainable harvest regime for Indonesia’s stony coral fishery with application to other coral exporting countries. in vol. 1692 1697 (Proceedings of 10th International Coral Reef Symposium, 2006).

[15] Pratchett, M. S. *et al. Vulnerability of commercially harvested corals to fisheries exploitation versus environmental pressures*. https://www.frdc.com.au/sites/default/files/products/2014-029-DLD.pdf FRDC (2020).

[16] Jones AM (2011). Raiding the coral nurseries?. Diversity.

[17] De’ath, G., Fabricius, K. E., Sweatman, H. & Puotinen, M. The 27-year decline of coral cover on the Great Barrier Reef and its causes. *Proc. Natl. Acad. Sci.* **109**, 17995–17999 (2012). 10.1073/pnas.1208909109 PMC3497744 23027961

[18] Pratchett MS (2020). Bleaching susceptibility of aquarium corals collected across northern Australia. Coral Reefs.

[19] DAWE. *Expert advice for the assessment of Australian coral fisheries – Queensland Coral Fishery 2006–2007 to 2019–2020* . https://www.agriculture.gov.au/sites/default/files/documents/qld-coral-expert-advice-assessment-australian-coral-fisheries-2021.pdf (2021).

[20] Morton, J., Jacobsen, I. & Dedini, E. *Queensland Coral Fishery Ecological Risk Assessment Update [Phase 1]*. (2022).

[21] Mellin, C. *et al. A standardised national assessment of the state of coral and rocky reef biodiversity*. https://www.nespmarine.edu.au/system/files/Mellin%20et%20al_D5_SS3_A%20standardised%20national%20assessment%20of%20the%20state%20of%20coral_Aug_21.pdf *Rep Nat. Enviro. Sci. Prog. MBH, UTAS* (2021).

[22] Ross MA (1984). A quantitative study of the stony coral fishery in Cebu Philippines. Mar. Ecol..

[23] Longenecker K, Bolick H, Langston R (2015). Estimating sustainable live-coral harvest at Kamiali wildlife management area Papua New Guinea. PLoS ONE.

[24] Pacey KI, Caballes CF, Pratchett MS (2022). Size-weight relationships for estimating harvestable biomass of *Acropora* corals on Australia’s Great Barrier Reef. Mar. Environ. Res..

[25] Mellin C (2019). Spatial resilience of the Great Barrier Reef under cumulative disturbance impacts. Glob. Change Biol..

[26] Penin L (2010). Early post-settlement mortality and the structure of coral assemblages. Mar. Ecol. Prog. Ser..

[27] Lirman D (2000). Fragmentation in the branching coral *Acropora palmata* (Lamarck): Growth, survivorship, and reproduction of colonies and fragments. J. Exp. Mar. Biol. Ecol..

[28] Smith LD, Hughes TP (1999). An experimental assessment of survival, re-attachment and fecundity of coral fragments. J. Exp. Mar. Biol. Ecol..

[29] Wallace CC (1985). Reproduction, recruitment and fragmentation in nine sympatric species of the coral genus *Acropora*. Mar. Biol..

[30] Harriott VJ (2003). Can corals be harvested sustainably?. Ambio.

[31] Dornelas M, Madin JS, Baird AH, Connolly SR (2017). Allometric growth in reef-building corals. Proc. R. Soc. B Biol. Sci..

[32] Pratchett, M. S. *et al.* Spatial, Temporal and Taxonomic Variation in Coral Growth—Implications for the Structure and Function of Coral Reef Ecosystems. in *Oceanography and Marine Biology* (eds. Hughes, R. N., Hughes, D. J., Smith, I. P. & Dale, A. C.) 224–305 (CRC Press, 2015).

[33] Cowman PF (2020). An enhanced target-enrichment bait set for Hexacorallia provides phylogenomic resolution of the staghorn corals (Acroporidae) and close relatives. Mol. Phylogenet. Evol..

[34] Bürkner, P.-C. Bayesian Distributional Non-Linear Multilevel Modeling with the R Package brms. *arXiv preprint: 1705.11123* (2017).

[35] R. Core Team. R: A Language and Environment for Statistical Computing. R Foundation for Statistical Computing (2022).

[36] Bürkner P-C (2017). brms: An R Package for Bayesian multilevel models using Stan. J. Stat. Softw..

[37] Vehtari A, Gelman A, Gabry J (2017). Practical Bayesian model evaluation using leave-one-out cross-validation and WAIC. Stat. Comput..

[38] Wickham, H. *ggplot2: Elegant Graphics for Data Analysis*. (Springer Cham, 2016).

[39] Schneider CA, Rasband WS, Eliceiri KW (2012). NIH Image to ImageJ: 25 years of Image Analysis. Nat. Methods.

