# Supplementary material for: Using size-weight relationships to estimate biomass of heavily targeted aquarium corals by Australia’s coral harvest fisheries
Source: Sci Rep. 2023 Jan 26;13:1448. doi: 10.1038/s41598-023-28447-w (PMC9879994; doi:10.1038/s41598-023-28447-w)
Supplement: Supplementary file 1 — Supplementary Information. [file 41598_2023_28447_MOESM1_ESM.docx]

**Appendix A.** Table showing estimate and standard error (*SE*) of each non-linear parameter constant, ‘*a*’ (i.e. scaling factor) and ‘*b*’ (i.e. exponent), along with lower (L) and upper (U) 95% Highest Density Intervals (HDIs) for each species. Estimates and *SE* were extracted using the ‘as_draws_rvars’ function of the ‘posterior’ package (Bürkner et al., 2021). HDIs were extracted using the ‘ci’ function of the package ‘bayestestR’ (Makowski et al., 2019).

| **Growth Form** | **estimate ± *SE*** | **L. 95% HDI** | **U. 95% HDI** |
| --- | --- | --- | --- |
| **Non-linear constant ‘*a*’** |  |  |  |
| *Catalaphyllia jardinei* | 0.69 ± 0.55 | 0.01 | 1.76 |
| *Duncanopsammia axifuga* | 0.20 ± 0.038 | 0.13 | 0.27 |
| *Euphyllia glabrescens* | 0.43 ± 0.11 | 0.25 | 0.67 |
| *Homophyllia australis* | 0.73 ± 0.14 | 0.48 | 1.01 |
| *Micromussa lordhowensis* | 1.83 ± 0.23 | 1.42 | 2.31 |
| *Trachyphyllia geoffroyi* | 0.36 ± 0.033 | 0.30 | 0.42 |
| **Non-linear constant ‘*b*’** |  |  |  |
| *Catalaphyllia jardinei* | 2.73 ± 0.35 | 2.09 | 3.41 |
| *Duncanopsammia axifuga* | 2.77 ± 0.06 | 2.65 | 2.89 |
| *Euphyllia glabrescens* | 2.74 ± 0.094 | 2.54 | 2.90 |
| *Homophyllia australis* | 2.59± 097 | 2.38 | 2.77 |
| *Micromussa lordhowensis* | 2.06 ± 0.045 | 1.97 | 2.15 |
| *Trachyphyllia geoffroyi* | 2.89 ± 0.037 | 2.82 | 2.97 |

**Appendix B.** Additional size-weight model parameters including seed, chains, cores, iterations (iter), warmup, thin, and table showing LPS coral species and model priors (where dist = distribuition, coef = coefficient, and nlpar = non-linear parameter).

All models run using seed = 1234, chains = 4, cores = 4, iter = 12000, warmup = 5000, thin = 12, distributional family = gaussian.

| **model** | **dist** | **prior** | **class** | **coef** | **nlpar** | | **source** |
| --- | --- | --- | --- | --- | --- | --- | --- |
| *Catalaphyllia jardinei* | normal | 0.5, 1.5 | b |  | | ‘a’ | user |
|  | normal | 0.5, 1.5 | b | Intercept | | ‘a’ | vectorized |
|  | normal | 3, 1 | b |  | | ‘b’ | user |
|  | normal | 3, 1 | b | Intercept | | ‘b’ | vectorized |
|  | Student_t | 3, 0, 45.2 | sigma |  | |  | default |
| *Duncanopsammia axifuga* | normal | 0.5, 0.3 | b |  | | ‘a’ | user |
|  | normal | 0.5, 0.3 | b | Intercept | | ‘a’ | vectorized |
|  | normal | 3, 0.4 | b |  | | ‘b’ | user |
|  | normal | 3, 0.4 | b | Intercept | | ‘b’ | vectorized |
|  | Student_t | 3, 0, 123.1 | sigma |  | |  | default |
| *Euphyllia glabrescens* | normal | 1.4, 0.5 | b |  | | ‘a’ | user |
|  | normal | 1.4, 0.5 | b | Intercept | | ‘a’ | vectorized |
|  | normal | 2.4, 0.6 | b |  | | ‘b’ | user |
|  | normal | 2.4, 0.6 | b | Intercept | | ‘b’ | vectorized |
|  | Student_t | 3, 0, 99.3 | sigma |  | |  | default |
| *Homophyllia australis* | normal | 1, 0.5 | b |  | | ‘a’ | user |
|  | normal | 1, 0.5 | b | Intercept | | ‘a’ | vectorized |
|  | normal | 2.3, 0.4 | b |  | | ‘b’ | user |
|  | normal | 2.3, 0.4 | b | Intercept | | ‘b’ | vectorized |
|  | Student_t | 3, 0, 34.1 | sigma |  | |  | default |
| *Micromussa lordhowensis* | normal | 1, 0.8 | b |  | | ‘a’ | user |
|  | normal | 1, 0.8 | b | Intercept | | ‘a’ | vectorized |
|  | normal | 2.5, 0.5 | b |  | | ‘b’ | user |
|  | normal | 2.5, 0.5 | b | Intercept | | ‘b’ | vectorized |
|  | Student_t | 3, 0, 207.6 | sigma |  | |  | default |
| *Trachyphyllia geoffroyi* | normal | 0.5, 0.1 | b |  | | ‘a’ | user |
|  | normal | 0.5, 0.1 | b | Intercept | | ‘a’ | vectorized |
|  | normal | 3, 0.1 | b |  | | ‘b’ | user |
|  | normal | 3, 0.1 | b | Intercept | | ‘b’ | vectorized |
|  | Student_t | 3, 0, 112.7 | sigma |  | |  | default |
| Species Comparison (“ANCOVA”) model | normal | 1.5, 0.8 | b |  | |  |  |
|  | normal | 1.5, 0.8 | b | Intercept | | ‘a’ | user |
|  | normal | 0, 1.6 | b |  | | ‘a’ | vectorized |
|  | normal | 0.45, 0.04 | b | Intercept | | ‘b’ | user |
|  | Student_t | 3, 0, 2.5 | sigma |  | | ‘b’ | vectorized |
|  | normal | 0, 1.6 | b | DMAX.cm | | ‘c’ | vectorized |
|  | normal | 0, 1.6 | b | DMAX.cm:speciesDuncanopsammiaaxifuga | | ‘c’ | vectorized |
|  | normal | 0, 1.6 | b | DMAX.cm:speciesEuphylliaglabrescens | | ‘c’ | vectorized |
|  | normal | 0, 1.6 | b | DMAX.cm:speciesHomophylliaaustralis | | ‘c’ | vectorized |
|  | normal | 0, 1.6 | b | DMAX.cm:speciesMicromussalordhowensis | | ‘c’ | vectorized |
|  | normal | 0, 1.6 | b | DMAX.cm:speciesTrachyphylliageoffroyi | | ‘c’ | vectorized |
|  | normal | 0, 1.6 | b | Intercept | | ‘c’ | vectorized |
|  | normal | 0, 1.6 | b | speciesDuncanopsammiaaxifuga | | ‘c’ | vectorized |
|  | normal | 0, 1.6 | b | speciesEuphylliaglabrescens | | ‘c’ | vectorized |
|  | normal | 0, 1.6 | b | speciesHomophylliaaustralis | | ‘c’ | vectorized |
|  | normal | 0, 1.6 | b | speciesMicromussalordhowensis | | ‘c’ | vectorized |
|  | normal | 0, 1.6 | b | speciesTrachyphylliageoffroyi | | ‘c’ | user |
|  | normal | 0.45, 0.04 | b |  | | ‘c’ | vectorized |
